# HIV knowledge and stigma among dietetic students in Indonesia: implications for the nutrition education system

**DOI:** 10.1186/s12879-020-05379-8

**Published:** 2020-09-09

**Authors:** Mutiara Tirta Prabandari Lintang Kusuma, Tandalayo Kidd, Nancy Muturi, Sandra B. Procter, Linda Yarrow, Wei-Wen Hsu

**Affiliations:** 1 Department of Nutrition and Health, Faculty of Medicine, Public Health, and Nursing, Universitas Gadjah Mada, Yogyakarta, Indonesia; 2 Department of Food, Nutrition, Dietetics, and Health, College of Health and Human Sciences, Kansas State University, Manhattan, Kansas USA; 3 A.Q. Miller School of Journalism and Mass Communications, College of Arts and Sciences, Kansas State University, Manhattan, Kansas USA; 4 Department of Statistics, College of Arts and Sciences, Kansas State University, Manhattan, Kansas USA

**Keywords:** HIV, AIDS, Attribution theory, Stigma, HIV knowledge, Nutrition education, Dietetic student

## Abstract

**Background:**

Studies have demonstrated that health care students and practitioners are not immune to stigma towards people living with HIV (PLHIV). This attitude could lead to poor quality of care if it remains uncorrected. However, little is known about dietetic students’ acceptance of PLHIV despite their substantial role in treatment. This study aimed to measure the extent of knowledge and stigma towards PLHIV among dietetic students and to determine the associated factors using the attribution theory.

**Methods:**

Students from three dietetics schools in Indonesia (*n* = 516) were recruited to participate in this cross-sectional study. Survey questions covered demographic information, interaction with PLHIV, access to information sources, cultural values, and beliefs as predictor variables. The outcome variables were comprehensive knowledge of HIV, HIV and nutrition-specific knowledge, and attitudes. Analyses with linear regression and the stepwise selection were performed to determine factors related to the outcome.

**Results:**

The levels of HIV comprehensive knowledge and HIV-nutrition specific knowledge among dietetic students were low, as indicated by the average score of 19.9 ± 0.19 (maximum score = 35) and 8.0 ± 0.11 (maximum score = 15), respectively. The level of negative attitudes towards PLHIV was high, with 99.6% of participants reported having a high stigma score. Types of university affiliation (public or private), beliefs and values, exposure to HIV discourse, access to printed media, and years of study were significantly related to HIV comprehensive knowledge (*p* < 0.05). Nutrition-specific knowledge was also correlated with university affiliation, beliefs and values, participation in HIV discussion, and years of study (*p* < 0.05). HIV comprehensive knowledge, university affiliation, discussion participation, and ethnicities were associated with attitudes (*p* < 0.05).

**Conclusions:**

Awareness and acceptance of PLHIV must be further improved throughout dietetic training to ensure patients’ quality of care since students represent future dietary care providers. Considering the consistent findings that affiliation to education institution correlates with HIV knowledge and attitude, some examinations concerning the curriculum and teaching conduct might be necessary.

## Background

Human Immunodeficiency Virus (HIV) remains a significant public health threat worldwide despite the current situation which shows improvement in the number of new infections and mortality rates. In Indonesia, it is estimated that about 630,000 people were living with HIV at the end of 2017. The data show that HIV incidence decreased from 0.26 to 0.19 between 2010 and 2017, but the mortality rate increased by approximately 69% [1].

HIV infection adversely affects individual nutrition status. People living with HIV (PLHIV) are more prone to rapid weight loss and malnutrition due to prolonged infection, eating difficulties, and metabolic alterations [2–5]. Nutrition problems associated with HIV and its treatment occur in most patients and could be indicative of the stage and progression of the infection. Research suggested that proper nutrition care is necessary to strengthen the immune system, delay the disease progression, and improve patients’ quality of life [4–7].

The fact that dietary management is considered as one key pillar in HIV treatment requires the ability of dietitians to transfer knowledge and skills to the patients through nutrition education and counseling [8–10]. However, despite the crucial role of dietitians, little is known about their awareness and ability in performing nutrition care for PLHIV as earlier studies were mostly done among other health providers [11–14]. Unlike doctors and nurses who have close contacts with patients, dietitians’ interaction is limited to counseling sessions [15]. This low personal contact with PLHIV may affect their self-awareness concerning prejudice and stigma.

Studies have demonstrated a wide range of levels of HIV-related knowledge and attitudes among healthcare students from different study populations. A study among medical and nursing students in Fiji showed adequate HIV knowledge and favorable attitudes towards HIV patients [16]. Similar results were reported among dental students in the United Arab Emirates [17]. However, a survey among pre-clinical students in Israel found that although their knowledge was relatively good, prejudicial attitudes remained prevalent, which was attributed to shame-related stigma and fear of contagions [18]. This finding corresponds with a study among Turkish nursing students in which the participants expressed hesitation to work with HIV patients due to misconceptions [19].

Acceptance of PLHIV in Indonesia is low in the community and also among health providers [11–13]. UNAIDS reported that the percentage of PLHIV who experienced discriminatory attitudes increased from 57.1 to 62.8% between 2007 and 2012 [1]. Previous studies also demonstrated a widespread stigmatized attitude among doctors, nurses, and midwives [11, 12, 20]. A study on HIV in hospitals and clinics in Aceh, Indonesia, found a high level of negative attitudes among health professionals, including dietitians. The stigma found in this study was classified as a value-driven stigma, which corresponded to the blame and shame prejudice. With this attitude, HIV is regarded as a ‘disease of a bad person’ and immoral; hence, how patients contracted the disease would determine acceptance. Consequently, low HIV testing and counseling uptake, delayed treatments, disengagement from health care, and poor adherence to medication among PLHIV in Indonesia have been widely reported [13, 21, 22].

Dietetic students represent the future of dietetic care, and their academic experiences will be reflected in their future practices. The chances are high that they will find themselves working in the field of HIV and AIDS and engage with the key populations. Therefore, dietetic schools are required to provide to the best of their ability the adequate knowledge and skills needed to provide care for patients with HIV as well as learning experiences that encourage acceptance and willingness to help the patients. Nonetheless, conducting courses about HIV and AIDS is not mandatory for any dietetic school in Indonesia.

This study aimed at measuring the level of knowledge (HIV comprehensive and nutrition-specific knowledge) and stigma towards PLHIV among dietetic students in Indonesia. The attribution theory used in this study is concerned with how common sense operates to explain events and the psychological consequences of such an attribution process, which may present a perceived causality instead of actual causality [23]. This theory is widely used in HIV studies to explain stigma [24]. Because this study is among the first to explore HIV knowledge and attitude among dietetic students in Indonesia, we believe the outcome can substantially contribute to improve the nutrition education system.

## Methods

### Study design

This research was a cross-sectional analytical study involving three dietetic schools in Indonesia and conducted between 2016 and 2017. Two hypotheses were tested: (1) there is an association between access to HIV-related information with knowledge and attitudes; and (2) there is an association between personal values and HIV knowledge and attitudes.

The ethical considerations of this study were fully approved by the Kansas State University Institutional Board Review (IRB) # 8555 and the Medical and Health Research Ethics Committee (MHREC) Faculty of Medicine Universitas Gadjah Mada – Dr. Sardjito General Hospital, reference number KE/FK/0129/EC/2017.

### Study setting

This study was conducted in three dietetic schools in Indonesia: two state universities and one private university. All universities are located in Java island, which has the largest population and highest proportion of HIV cases [25]. Two universities are in Jogjakarta province and one is in Central Java province, both are approximately 500 km away from Jakarta, the capital city of Indonesia. Both provinces experience increased incidence and prevalence of HIV and AIDS over the years, with Central Java province reporting higher prevalence compared to Jogjakarta province [25]. All universities are affiliated with teaching hospitals that are appointed as HIV referral hospitals. To control the spread of HIV, the Government of Indonesia has appointed 278 referral hospitals throughout the country, under the Indonesian Ministry of Health Decree number 782/MENKES/SK/IV/2011. All HIV-suspected patients are referred to these hospitals to get tested, counseling, and treatment.

### Study participants

The study population consisted of junior and senior dietetic students who were selected considering their progression through the curriculum. The sample size was determined through total population sampling with the average participation rate of 87.5% from all participating institutions. The sample was comprised of 516 students from two state universities (n_1_ = 126 and n_2_ = 183) and one private university (*n* = 207). Before signing the written consent form, participants were informed that participation was voluntary; they could skip any questions and drop out of the study at any time; their answers would remain anonymous and would not affect their academic standing.

### Instruments

The questionnaire consisted of eight sections: 1) sociodemographic, 2) interaction with PLHIV, 3) access to information and social media, 4) Asian Value Scales, 5) Beliefs and Value Scales, 6) HIV comprehensive knowledge, 7) HIV and nutrition-specific knowledge, and 8) attitudes. The Asian Value Scale (AVS) and the Beliefs and Values Scales (BVS) had been used in previous studies, while the other questionnaires were developed prior to this study [26]. All questionnaires were translated into the Indonesian Language and tested for readability as well as internal consistency before the data collection began. Questionnaire testing was performed among thirty students from other universities with similar characteristics (age, years of study, and academic background). We used Cronbach’s alpha and Kuder Richardson-20 to measure internal consistency (reliability). Table 1 provides information on the questionnaires, constructs, and results of the test.

**Table 1:** Summary of study instruments

Section	Definition and constructs	Internal consistency
Sociodemographic	Age, gender, years of study, ethnic and family background, religion, and marital status	–
Interaction with PLHIV	Ever known of someone who has HIV, ever participated in HIV courses or classroom discussion, ever participated in HIV training, and awareness of HIV-related guidelines or protocols	–
Access to information and media	The estimated time spent on media and social media (TV, radio, internet, newspaper/magazines, Facebook, and Twitter).	
Asian Value Scales	Adherence to Asian culture; conformity to norms, family recognition through achievement, emotional self-control, collectivism, humility, and filial piety.	Cronbach’s alpha = 0.898
Beliefs and Values Scales	Assessing the level of religiosity and spirituality among study participants	Cronbach’s alpha = 0.859
HIV-comprehensive knowledge	Basic knowledge of HIV and AIDS; modes of transmission, prevention method, treatment, and human rights.	Kuder-Richardson (KR)-20 = 0.762
HIV-nutrition specific knowledge	Nutrition care process (NCP) for HIV patients; nutrition assessment, diagnosis, intervention, monitoring and evaluation, counseling, as well as infant and young child feeding practices	
Attitudes	Attitudes towards PLHIV measured through agreements with sixteen statements, which include willingness to treat PLHIV (8 items), personal responsibility or blame (1 item), perceived dangerousness and fear (4 items), controllability (1 item), and respect patients’ rights (2 items)	Cronbach’s alpha = 0.763

The previous test on the AVS questionnaire showed that the alpha coefficients for each construct were low; hence, only the total score was suggested for the final analysis [26]. On the other hand, the previous test on the BVS questionnaire demonstrated high internal and test-retest reliability across various religious and non-religious standpoints [27]. Item responses in the knowledge questionnaire were coded as ‘Yes,’ ‘No’ and ‘Do Not Know,’ with the response of ‘Do Not Know’ scored as incorrect. Higher scores indicated higher levels of HIV knowledge. The correct responses from 35 items were combined to yield a single HIV comprehensive knowledge score. The HIV and nutrition-specific knowledge scores were used as a subscale containing 15 questions and were treated as such in the subsequent statistical analysis. Participants who provided more than 75% of correct answers were considered to have adequate knowledge.

The attitude questionnaire was developed in accordance with the previous study that tested the attribution of stigma in ten diseases. The study found that stigma towards PLHIV had specific attribution properties, which emphasized the aspects of responsibility, blame, and pity [24]. Our questionnaire consisted of 16 items that were measured using a Likert scale, ranging from 1 (strongly disagree) to 5 (strongly agree); hence, the maximum total score was 80. The cutoff to determine favorable attitude was 20, in which, the higher score was equal with less favorable or stigmatized attitudes.

### Statistical analysis

The data were analyzed using SPSS 21.0 (SPSS Inc., Chicago, IL, USA, 2012). The mean and standard error of the mean (SE) were used to describe the continuous variables, whereas the proportion was used to describe categorical variables. Differential analysis between the state and private dietetic schools was performed using independent t-test for the continuous variables and Chi-squared test for the categorical variables. No missing values were found in this study. The linear regression test was conducted since the outcome variables were retained in the ratio scale and the stepwise selection method was applied to obtain the best-fit model for the study outcomes. Prior to fitting the regression model, we tested the model assumption to check if the residuals met the criteria, and there seemed to be no clear violation of the model assumptions. The level of statistical significance was determined at *p* < 0.05 for all tests.

## Results

### Characteristics of study participants

Of the 516 students participating in this study, 94.0% were female, 59.9% studied in a state university, and 74.8% were Muslim. The mean of participants’ age was 20.7 years (SE ± 0.04). We found that students from the private university were significantly more varied in terms of their ethnic background, religious practice, and domicile of origin than those from the state universities (Table 2).

**Table 2:** Sociodemographic characteristics of the study participants

Variables	State University (*N* = 309)	Private University (*N* = 207)	Total (*N* = 516)
Age (years, mean ± SE)	20.7 ± 0.04	20.7 ± 0.07	20.7 ± 0.04
Gender (n, %)			
Male	15 (4.9)	16 (7.7)	31 (6.0)
Female	294 (95.1)	191 (92.3)	485 (94.0)
College year (n, %)			
Junior	164 (53.1)	107 (51.7)	271 (52.5)
Senior	145 (46.9)	100 (48.3)	245 (47.5)
Ethnicity (n, %) *			
Java	239 (77.3)	81 (39.1)	320 (62.0)
Non-Java	70 (22.7)	126 (60.9)	196 (38.0)
Religion (n, %) *			
Islam	257 (83.2)	129 (62.3)	386 (74.8)
Non-Islam	52 (16.8)	78 (37.7)	130 (25.2)
Marital status (n, %)			
Married	1 (0.3)	2 (1.0)	3 (0.6)
Single / not married	308 (99.7)	205 (99.0)	513 (99.4)
Domicile of origin (n, %) *			
Urban	182 (58.9)	103 (49.7)	285 (55.2)
Sub-urban	62 (20.1)	36 (17.4)	98 (19.0)
Rural	65 (21.0)	68 (32.9)	133 (25.8)

*n* number of participants, *SE* standard error of the mean

*statistically different at *p* < 0.05 (comparisons between university groups with Chi-squared test)

Data presented in Table 3 show that most participants had no prior interaction with any PLHIV (77.1%) and never participated in HIV training (69.8%). Most of them had participated in HIV discussion or courses (79.7%), yet, only a few of them were aware of HIV-related guidelines and protocols (36.0%). Students from the private university were found to have higher opportunities to take part in HIV discourse and undergo training. Likewise, they were more aware of the HIV-related guidelines and protocols (*p* < 0.05). We also found that most participants obtained information from the television (94.9%) and Internet (95.7%), and spent a significant amount of time accessing social media.

**Table 3:** Experience with PLHIV and access to information

Variables	State University (*N* = 309)	Private University (*N* = 207)	Total (*N* = 516)
Ever known of PLHIV (n, %)			
Yes	77 (24.9)	41 (19.8)	118 (22.9)
No	232 (75.1)	166 (80.2)	398 (77.1)
Ever discussed HIV in class (n, %) *			
Yes	223 (72.2)	188 (90.8)	411 (79.7)
No	86 (27.8)	19 (9.2)	105 (20.3)
Participated in HIV training (n, %) *			
Yes	44 (14.2)	112 (54.1)	156 (30.2)
No	265 (85.8)	95 (45.9)	360 (69.8)
Aware of HIV protocols (n, %) *			
Yes	83 (26.9)	103 (49.8)	186 (36.0)
No	226 (73.1)	104 (50.2)	330 (64.0)
Use of TV per day (n, %) *			
Never	13 (4.2)	13 (6.3)	26 (5.1)
≤ 1 h	120 (38.8)	51 (24.6)	171 (33.1)
2–3 h	115 (37.2)	84 (40.6)	199 (38.6)
4–5 h	37 (12.0)	29 (14.0)	66 (12.8)
6–7 h	18 (5.8)	11 (5.3)	29 (5.6)
> 7 h	6 (2.0)	19 (9.2)	25 (4.8)
Use of radio per day (n, %) *			
Never	96 (31.1)	99 (47.8)	195 (37.8)
≤ 1 h	157 (50.8)	81 (39.1)	238 (46.1)
2–3 h	44 (14.2)	14 (6.8)	58 (11.2)
4–5 h	9 (2.9)	7 (3.4)	16 (3.1)
6–7 h	2 (0.7)	2 (1.0)	4 (0.8)
> 7 h	1 (0.3)	4 (1.9)	5 (1.0)
Use of internet per day (n, %) *			
≤ 1 h	5 (1.6)	17 (8.2)	22 (4.3)
2–3 h	36 (11.7)	55 (26.6)	91 (17.6)
4–5 h	91 (29.4)	46 (22.2)	137 (26.6)
6–7 h	63 (20.4)	31 (15.0)	94 (18.2)
> 7 h	114 (36.9)	58 (28.0)	172 (33.3)
Use of printed media per day (n, %)			
Never	84 (27.2)	66 (31.9)	150 (29.1)
≤ 1 h	191 (61.9)	112 (54.1)	303 (58.7)
2–3 h	30 (9.7)	23 (11.1)	53 (10.3)
4–5 h	2 (0.6)	0 (0.0)	2 (0.4)
6–7 h	2 (0.6)	5 (2.4)	7 (1.3)
> 7 h	0 (0.0)	1 (0.5)	1 (0.2)
Access to Facebook per day (n, %) *			
Never	47 (15.2)	25 (12.1)	72 (14.0)
≤ 1 h	206 (66.7)	81 (39.1)	287 (55.6)
2–3 h	37 (12.0)	60 (29.0)	97 (18.8)
4–5 h	13 (4.2)	20 (9.7)	33 (6.4)
6–7 h	5 (1.6)	12 (5.8)	17 (3.3)
> 7 h	1 (0.3)	9 (4.3)	10 (1.9)
Access to Twitter per day (n, %) *			
Never	118 (38.2)	109 (52.6)	227 (44.0)
≤ 1 h	150 (48.5)	71 (34.3)	221 (42.8)
2–3 h	25 (8.1)	20 (9.7)	45 (8.7)
4–5 h	10 (3.2)	4 (1.9)	14 (2.7)
6–7 h	4 (1.3)	1 (0.5)	5 (1.0)
> 7 h	2 (0.7)	2 (1.0)	4 (0.8)

*n* number of participants

*statistically different at *p* < 0.05 (comparisons between university groups with Chi-squared test)

### Cultural values and spirituality

The mean AVS score of the participants was 4.9 (SE ± 0.02) out of 7.0 considered as high adherence to the Asian values [28]. The AVS subscales were calculated to provide a clear depiction of the tenets of the Asian philosophy; however, these subscales were excluded from further analysis considering the low alpha coefficient [26]. Similarly, with an average score of 58.2 (SE ± 0.36) on the BVS test, the participants showed a high level of spirituality (97.3%) [27]. There were no significant differences in the BVS and AVS scores between students from the public and private dietetic schools (Table 4).

**Table 4:** Social, cultural, and spiritual characteristics of the study participants

Variables	State University (*N* = 309)	Private University (*N* = 207)	Total (*N* = 516)
Asian Values Scales (AVS)			
Total scales (mean ± SE)	4.9 ± 0.03	4.8 ± 0.04	4.9 ± 0.02
Conformity to norms	4.7 ± 0.03	4.7 ± 0.04	4.7 ± 0.02
Family recognition	4.6 ± 0.06	4.8 ± 0.09	4.7 ± 0.05
Emotional self-control *	5.1 ± 0.04	4.9 ± 0.06	5.0 ± 0.03
Collectivism *	5.2 ± 0.04	4.9 ± 0.08	5.0 ± 0.04
Humility *	5.4 ± 0.05	5.2 ± 0.07	5.3 ± 0.04
Filial piety *	4.6 ± 0.04	4.7 ± 0.05	4.6 ± 0.03
Beliefs and Values Scales (BVS)			
Total scales (mean ± SE)	58.7 ± 0.42	57.5 ± 0.65	58.2 ± 0.36
Low (n, %)	5 (1.6)	9 (4.3)	14 (2.7)
High (n, %)	304 (98.4)	198 (95.7)	502 (97.3)

*n* number of participants, *SE* standard error of the mean

*statistically different at *p* < 0.05 (comparisons between university groups with t-test)

### HIV knowledge and attitudes

The mean and SE for the HIV comprehensive and nutrition-specific knowledge were 19.9 ± 0.19 and 8.0 ± 0.11, respectively. With the cutoff of 75% for all composite scores, we found that less than 3% of the participants had adequate knowledge of the standardized nutrition care for HIV patients. Similarly, the mean and SE of the attitudes score was 42.7 ± 0.27, which showed that only 0.4% of the respondents had positive attitudes towards PLHIV. We found no significant differences in scores of knowledge and attitudes between students from private and state dietetics schools as presented in Table 5.

**Table 5:** HIV knowledge and attitudes among study participants

Variables	State University (*N* = 309)	Private University (*N* = 207)	Total (*N* = 516)
HIV comprehensive knowledge (mean ± SE)	21.3 ± 0.21	18.1 ± 0.33	19.9 ± 0.19
Low (n, %)	299 (96.8)	204 (98.6)	503 (97.5)
High (n, %)	10 (3.2)	3 (1.4)	13 (2.5)
HIV-nutrition knowledge (mean ± SE)	8.5 ± 0.13	7.3 ± 0.18	8.0 ± 0.11
Low (n, %)	302 (97.7)	202 (97.6)	504 (97.7)
High (n, %)	7 (2.3)	5 (2.4)	12 (2.3)
HIV stigma (mean ± SE)	41.2 ± 0.32	44.9 ± 0.43	42.7 ± 0.27
Not favorable (n, %)	309 (100.0)	205 (99.0)	514 (99.6)
Favorable (n, %)	0 (0.0)	2 (1.0)	2 (0.4)

*n* number of participants, *SE* standard error of the mean

*statistically different at *p* < 0.05 (comparisons between university groups with t-test)

### Factors associated with HIV knowledge and stigma

Using the attribution theory, we studied the factors associated with HIV knowledge and attitudes. The factors being tested were sociodemographic characteristics, interaction with key populations, participation in training, exposure to HIV discourse in the classroom setting, access to information, adherence to Asian values, and spirituality. The variable of HIV comprehensive knowledge was included in the linear regression test for the outcome variable of attitudes.

Table 6 presents the selected regression model for HIV knowledge and attitude; sub-analyses were performed to create a model to explain the nutrition-specific knowledge variable. Six predictor variables were retained in the HIV comprehensive knowledge model: types of university affiliation, beliefs and values, interaction with PLHIV, access to printed media, years of study, and participation in HIV discussion (adjusted R^2^ = 0.199, F = 22.294, *p* < 0.001). For the subset analysis, the model contained university affiliation, spirituality, discussion participation and years of study as predictor variables (adjusted R^2^ = 0.098, F = 15.023, *p* < 0.001). Moreover, HIV comprehensive knowledge, university affiliation, participation in HIV discourse and ethnicity were found to correlate with attitudes (adjusted R^2^ = 0.163, F = 26.021, *p* < 0.001).

**Table 6:** Results of linear regression analysis of knowledge and attitude towards PLHIV

	*B*	SE	β	*t*	*p*
**Outcome variable: HIV comprehensive knowledge**					
Constant	15.360	1.329		11.560	<.001
Type of university	−3.186	.369	−.353	−8.641	<.001
Beliefs and values scale	.117	.021	.218	5.483	<.001
Interaction with PLHIV	−.962	.435	−.091	−2.211	.028
Access to printed media	.514	.238	.086	2.156	.032
Years of study	−.776	.362	−.088	−2.145	.032
Ever discussed HIV in class	−.905	.451	−.082	−2.004	.046
Adjusted R^2^ = 0.199, *F* = 22.294, *p* < 0.001					
**Outcome variable: HIV-nutrition knowledge**					
Constant	6.218	.757		8.210	<.001
Type of university	−1.277	.217	−.253	−5.870	<.001
Beliefs and values scale	.047	.013	.155	3.692	<.001
Ever discussed HIV in class	−.884	.265	−.144	−3.340	.001
Years of study	−.444	.208	−.090	−2.131	.034
Adjusted R^2^ = 0.098, *F* = 15.023, *p* < 0.001					
**Outcome variable: HIV-related stigma**					
Constant	49.645	1.336		37.173	<.001
HIV comprehensive knowledge	−.361	.059	−.264	−6.085	<.001
Type of university	2.723	.593	.220	4.595	<.001
Ever discussed HIV in class	−1.782	.629	−.118	−2.835	.005
Ethnicity	−1.201	.547	−.096	−2.196	.029
Adjusted *R*^*2*^ = 0.163, *F* = 26.021, *p* < 0.001					

## Discussion

According to the attribution theory, the problems of HIV stigma and discrimination can stem from both internal and external factors [23]. Our study supports this theory as we found that HIV knowledge and attitudes were determined by several external factors such as academic experience, interaction with key populations, and access to media; and these were on top of the internal factors such as beliefs and cultural background. This study is the first to report on HIV knowledge and attitudes among dietetic students in Indonesia. Our findings show that the level of HIV comprehension was low and stigmatized attitudes were high, which have been observed elsewhere among other health care students [17–19, 29].

Dietitians and dietetic students are often overlooked in the discussion about HIV despite their critical roles in HIV care and control. Training is mostly provided to doctors and nurses who are considered to have higher responsibilities in treatment and at the same time more vulnerable due to their frequent contact with the patients [30]. Even when it is provided, topics delivered during the training were also limited to the technical or clinical necessities and often missed the more fundamental aspect of building a good relationship between patients and providers [31]. The Indonesian National AIDS Commission highlighted the need to equip HIV training for health professionals with the principles of effective service delivery, anti-discriminatory behavior, and human rights [32].

Similar to the medical schools, all dietetic schools in Indonesia follow the national curriculum guideline [33]. The Indonesian Nutrition Science Collegium and The Indonesian Association of Nutrition Academic Institution developed a dietetic curriculum in Indonesia. The curriculum is designed according to an established set of standard professional competencies and should be followed by all dietetic schools in Indonesia. However, each university can develop electives and courses that reflect their academic mission, yet courses in HIV and AIDS are not mandatory. This gap explains our findings that although most participants have enrolled in a class that discussed HIV, their understanding of the topic remains low. When HIV becomes an optional topic in the curriculum, there is no guarantee that students would receive adequate information about HIV which neither builds the level of professional competency needed to treat the patients nor cultivates non-prejudice attitudes.

Of special concern, the regression analysis consistently presented that university affiliation was related to knowledge and attitude towards PLHIV. Comparative analysis also revealed that students from the private university scored lower than those from the state university in all types of tests. The apparent difference in the way dietetic schools teach HIV to their students triggers discussion of what is being taught in each institution and requires further investigation considering the scarcity in the literature on nutrition education in Indonesia. Some assumptions might be taken from the medical education system. Private medical schools are known to have less bureaucratic burdens and more liberty to adopt innovations in exchange for reduced governmental subsidies. However, the lack of government control is likely to hamper the quality of education as it has been reported that the newer and smaller private institutions are having trouble in establishing an appropriate quality assurance system and accreditation [33–36].

Enforcing all dietetic schools to follow the same set of regulations for curriculum implementation and quality assurance might be necessary to ensure students having similar academic experiences and developing the skills needed in the workforce. Educational innovation and experimentation must be reported and documented properly, thus, allowing replication and comparative analysis [34]. Our study suggests that to improve HIV knowledge and attitude, dietetic schools need to ensure that students have opportunities to interact with the patients or key populations, have access to accurate information sources and discuss HIV in a class setting with their peers and instructors. These findings correspond with previous studies, which indicated higher exposure to HIV information and key populations might improve awareness and acceptance of the individuals [12, 29, 37].

Creating a safe, secure, and supportive learning environment is important to encourage an open and honest discussion about HIV. As suggested in our study, in a conservative country like Indonesia where HIV discussion tends to attract controversy, understanding the interlinking of religion, culture, sexuality, and HIV is important. A study in Bangladesh proposed the need to provide training to the teachers in addition to efforts in improving the curriculum. Through training, teachers can develop confidence and skills in imparting HIV knowledge with greater sensitivity, and thus inspire more positive attitudes among students [38, 39].

We defined stigma in this study as any type of negative attitude and misconduct performed by health professionals towards PLHIV due to their HIV status. However, stigma is a multifaceted problem, not one rooted in a simple causality. The attribution theory lends credence to the explanation that the process of attributing stigma usually happens without the person’s awareness [40]. Considering the tenets of the attribution theory that underpin the attributes given to PLHIV: personal responsibility, controllability, perceived dangerousness, and familiarity, an effort beyond an instructional approach is much-needed. Additionally, behavioral studies found that HIV-related stigma is particularly fluid compared to other diseases. The perceived dangerousness, for example, is amenable to following swift advancement in treatment and control [24]. Therefore, several studies suggested the need to conduct multilayered intervention targeting factors at the individual, interpersonal, community, institution, and structural levels to reduce stigma [41, 42].

Finally, addressing the stigma towards PLHIV among health professionals is important to prevent further public health consequences. Stigma in health facilities disrupts patients-providers communication that might lead to distrust and poor adherence to medication. It might also deter patients from accessing proper care in a timely manner, counseling and testing services as well as other harm reduction interventions [13, 21, 43–45]. Reducing stigma matters because it also has the potential to open further academic discussion and debate.

### Limitations

Several limitations should be taken into consideration in interpreting the results of this study. Firstly, the sample of dietetic students was selected conveniently from three universities only. Although these universities had wide coverage of student intake throughout Indonesia, which enables a culturally diverse study sample and might be typical of dietetic students in Indonesia, this assumption was not validated in the analysis. Nonetheless, the limited study setting might affect the external validity of the study and inferences must be made with caution to the study setting.

Secondly, the survey instruments might influence responses from the participants due to the use of standard Indonesian language and dialect, which may not address the discrepancies in language comprehension from participants who come from remote areas of Indonesia. As disparity in the quality of education is evident throughout Indonesia, students coming from marginalized areas might have trouble understanding the few jargons used in the questionnaire, despite our effort to use plain language. All this might explain the result of the reliability test of the instruments. Thirdly, considering the sensitivity of the issues, there is a risk of social desirability bias in the responses causing stigma to be underreported. However, the use of a self-administered questionnaire that gives more freedom and privacy to participants might minimize the problem. Finally, considering the nature of the cross-sectional study, our findings can only suggest an association with HIV knowledge and stigma.

## Conclusions

HIV-related knowledge among Indonesian dietetic students is low while the prejudicial attitudes are inappropriately high. Findings in our study also correspond with the attribution theory and provide evidence to our hypotheses that environmental factors determined HIV knowledge and attitudes as much as personal values. The differential findings between universities and lack of positive result in the cognition area despite the participation in HIV discourse, underline the need for further analysis on the education system and curriculum of dietetic schools. Evaluation of the teaching conduct might be necessary to reveal the learning process in each institution that may or may not dispel stigma among the students.

The findings of this study recommend two opportunities for intervention. First, given students are generally lacking in proper and formal training about HIV, curriculum improvement needs to be conducted systematically. This could be done by either strengthening the current curriculum with HIV discussion in all relevant courses or developing electives or mandatory courses on HIV and nutrition. Whichever options are deemed more viable, they should ensure enough room to discuss HIV beyond the clinical aspects. Since personal beliefs heavily influence HIV knowledge and attitudes, students need to be equipped with the opportunity to analyze and discuss their beliefs and values of HIV so that any potential prejudice can be mitigated. Second, considering high access to media and its influence on HIV awareness, social campaign activities are necessary to foster positive attitudes and acceptance of PLHIV, which calls for a multilayered approach. This type of intervention requires partnerships with cultural and religious experts.

## Data Availability

All data generated or analyzed during this study are included in this published article.

## Abbreviations

HIV: Human Immunodeficiency Virus
AIDS: Acquired Immune Deficiency Syndrome
PLHIV: People Living With HIV
SD: Standard deviation
SE: Standard error of the mean
AVS: Asian Values Scales
BVS: Beliefs and Values Scales
